# Draft genome sequence of a multidrug-resistant *Escherichia coli* MTRBAS03 strain isolated from feed trough of a dairy farm in Bangladesh

**DOI:** 10.1128/mra.00494-26

**Published:** 2026-08-21

**Authors:** Md. Makshuder Rahman Zim, Md. Abdullah Evna Hasan, Md. Monirul Islam, Anindita Ash Prome, Sadia Afrin Punom, Md. Bahanur Rahman, Md. Shafiqul Islam, Md. Tanvir Rahman

**Affiliations:** 1 Department of Microbiology and Hygiene, Faculty of Veterinary Science, Bangladesh Agricultural University 54492, Mymensingh, Bangladesh; Fluxus Inc., Sunnyvale, California, USA

**Keywords:** whole-genome sequencing, *Escherichia coli*, multidrug resistance (MDR), dairy farm, Bangladesh

## Abstract

We present the draft genome sequence of an *Escherichia coli* strain MTRBAS03, identified in a feed trough sample from a local dairy farm in Mymensingh City, Bangladesh. The genome spans 4,626,877 base pairs, 52 predicted antimicrobial resistance genes, 57 virulence genes, and a pathogenicity score of 0.977.

## ANNOUNCEMENT

Dairy farms are potential sources of bacteria, posing a public health concern (1). Here, we present the draft genome sequence of a multidrug-resistant (MDR) *Escherichia coli* strain isolated from a dairy farm.

In September 2025, three feed trough samples were aseptically collected from a dairy farm in Sadar upazila, Mymensingh (24.7425°N, 90.4168°E), transferred to 5 mL of nutrient broth, and incubated aerobically at 37°C overnight. The culture was streaked onto Eosin Methylene Blue agar (HiMedia, India) and incubated aerobically at 37°C overnight. A single dark, blue-black colony with a distinct green metallic sheen was selected and purified. The detection of *E. coli* was confirmed by PCR targeting *malB* gene and commercial matrix-assisted laser desorption/ionization time-of-flight mass spectrometry (MALDI-TOF) (2). The strain was confirmed as MDR using the Kirby-Bauer disk diffusion test (3) on Muller-Hinton agar media following standard protocol, in which ampicillin, amikacin, ciprofloxacin, imipenem, meropenem, doripenem, nalidixic acid, ceftriaxone, nitrofurantoin, and co-trimoxazole were tested, and ampicillin, amikacin, ceftriaxone, and nalidixic acid showed antimicrobial resistances. For whole-genome sequencing, the isolate was cultured aerobically at 37°C overnight in Luria-Bertani broth (BD, USA), and DNA was extracted using the DNeasy Blood & Tissue Kit (Qiagen, Germany). The quality and quantity of genomic DNA were determined by Bioanalyzer tools (4). The DNA library was prepared with the Illumina DNA Prep Reagent Kit (Illumina, USA), and sequencing was performed on the Illumina NextSeq 2000 platform, generating paired-end reads of 2 × 150 bp. Illumina adapters were trimmed with FastP v0.23.4 (5), quality assessed using FastQC v0.12.1 (6), genome assembly conducted using SPAdes v3.15.5 (7), and genome annotation was performed with the NCBI Prokaryotic Genome Annotation Pipeline (PGAP) v.6.10 (8). Taxonomic classification and contamination screening were completed using Kraken2 v2.1.3 (9), and genome completeness was evaluated with CheckM v1.2.3 (10). Sequence type was predicted using MLST v2.11 (https://github.com/tseemann/mlst), and virulence factor genes (VFGs) were identified using the virulence factor database (11) via ABRicate (https://github.com/tseemann/abricate). The pathogenicity index, plasmids, antibiotic resistance genes (ARGs), bacteriocin gene clusters, prophages, and metabolic subsystems were predicted using PathogenFinder v2.0.0 (12), PlasmidFinder v2.1 (13), the comprehensive antibiotic resistance database with RGI main v.6.0.2 (14), BAGEL4 (15), PHASTEST v1.0.1 (16), and RAST v2.0 (17), respectively. Unless otherwise noted, default parameters were used for all software.

The genome characteristics of the *E. coli* MTRBAS03 strain are given in Table 1. The closest *Escherichia coli* strain 19KM2415N was identified (CP151042). This genome comprises 4,626,877 bp. *De novo* assembly produced an N50 of 217,630 bp. The genome exhibits an average nucleotide identity of 99.26% compared to the reference genomes of *E. coli*. The genome was identified as sequence type ST5073 using seven-gene multilocus sequence typing (*adk*, *fumC*, *gyrB*, *icd*, *mdh*, *purA*, and *recA* genes). Furthermore, the genome contained no plasmids, 52 predicted ARGs, 57 predicted VFGs, 375 subsystems, 2 prophages, and 1 bacteriocin. The strain had a high predicted pathogenicity score of 0.977, matching 648 known pathogenic families, indicating significant potential for virulence in both animals and humans.

**TABLE 1:** Genomic features of the *E. coli* strain MTRBAS03 isolated from dairy farm

Attributes	Values
BioProject accession	PRJNA1437075
GenBank accession	JBWCAR000000000
BioSample accession	SAMN56493995
SRA accession	SRS28391837
Assembled genome size (bp)	4,645,806
Guanine–cytosine %	51%
Genome completeness (%)	99.47%
Number of raw reads (total sequences)	717,384
Sequence type	ST5073
N50 value (bp)	217,630
Number of plasmids	0
Number of contigs	139
Genome coverage (depth)	61.69×
Genes (total)	4,587
CDSs (total)	4,463
Genes (coding)	4,350
Genes (RNA)	124
rRNAs	7, 7, and 18 (5S, 16S, and 23S)
tRNAs	82
ncRNAs	10
Number of ARGs	52
Number of VFGs	57
Pathogenicity score	0.977
Matched with pathogenic families	648
Average nucleotide identity (ANI)	99.26%
Reference genome (RefSeq)	GCF_056175515.1-RS_2026_03_22
Number of intact prophages	2
Number of bacteriocins	1
Contamination	0.30%

Antibiotics	Inhibition zone stats (mm)
Ampicillin	00
Amikacin	10
Ceftriaxone	09
Nalidixic acid	00

## Data Availability

All the data and accession numbers are available in Table 1.
